# Spectromicroscopy of C_60_ and azafullerene C_59_N: Identifying surface adsorbed water

**DOI:** 10.1038/srep35605

**Published:** 2016-10-17

**Authors:** Dogan Erbahar, Toma Susi, Xavier Rocquefelte, Carla Bittencourt, Mattia Scardamaglia, Peter Blaha, Peter Guttmann, Georgios Rotas, Nikos Tagmatarchis, Xiaohui Zhu, Adam P. Hitchcock, Chris P. Ewels

**Affiliations:** 1 Institut des Matériaux Jean Rouxel, Université de Nantes, CNRS, Nantes, France; 2 Physics Department, Gebze Technical University, Gebze, Turkey; 3 University of Vienna, Faculty of Physics, Boltzmanngasse 5, A-1090 Vienna, Austria; 4 Institut des Sciences Chimiques de Rennes, UMR 6226 CNRS, Université de Rennes 1, Rennes, France; 5 Chemistry of Interaction Plasma-Surface (ChIPS), University of Mons, Mons, Belgium; 6 Institute for Materials Chemistry, TU Vienna, A-1060 Vienna, Austria; 7 Helmholtz-Zentrum Berlin für Materialien und Energie GmbH, Institute for Soft Matter and Functional Materials, Berlin, Germany; 8 Theoretical and Physical Chemistry Institute, National Hellenic Research Foundation, 48 Vassileos Constantinou Avenue, 11635 Athens, Greece; 9 Dept. of Chemistry and Chemical Biology, McMaster University, Hamilton, ON, L8S 4M1, Canada

## Abstract

C_60_ fullerene crystals may serve as important catalysts for interstellar organic chemistry. To explore this possibility, the electronic structures of free-standing powders of C_60_ and (C_59_N)_2_ azafullerenes are characterized using X-ray microscopy with near-edge X-ray adsorption fine structure (NEXAFS) spectroscopy, closely coupled with density functional theory (DFT) calculations. This is supported with X-ray photoelectron spectroscopy (XPS) measurements and associated core-level shift DFT calculations. We compare the oxygen 1*s* spectra from oxygen impurities in C_60_ and C_59_N, and calculate a range of possible oxidized and hydroxylated structures and associated formation barriers. These results allow us to propose a model for the oxygen present in these samples, notably the importance of water surface adsorption and possible ice formation. Water adsorption on C_60_ crystal surfaces may prove important for astrobiological studies of interstellar amino acid formation.

Membrane production is considered to be one of the critical steps in the formation of amino acids in the interstellar medium, and ultimately to the earliest stages of life^1^. However, the molecular interactions between carbon, nitrogen and oxygen in space remain enigmatic. Recent studies from the Spitzer radio telescope^2^ have identified large amounts of crystalline C_60_ in interstellar outflows. Midinfrared bands at 7.0, 8.5, 17.4 and 19.0 μm match those for neutral C_60_^3^,^4^, which has been suggested as a candidate for templating simple nucleobases in interstellar environments^5^. C_60_ crystal surfaces are a potential interstellar reaction membrane candidate; thus detailed characterization of the surface chemistry of fullerene crystal surfaces is required. However, quantitative analysis of spectroscopy data is complicated due to the potential ambiguity caused by carbonaceous impurities. For this reason it would be advantageous to also consider fullerene structures containing chemical markers, such as heterofullerenes.

Amongst heterofullerenes, the best-known and most studied species are the nitrogen-substituted azafullerenes, notably the mono aza[60]fullerene (C_59_N)^6^,^7^,^8^,^9^,^10^,^11^,^12^,^13^,^14^, where a single carbon atom is replaced by nitrogen. The resultant carbon radical is stabilized by forming an intermolecular sp^3^-like bond with a similar radical carbon in a neighboring azafullerene, with a predicted binding energy on the order of 0.65 eV^15^. Thus the preferred structure at room temperature or below is the (C_59_N)_2_ dimer (Fig. 1)^16^. Little is known about the spontaneous oxidation behavior of azafullerenes as compared to C_60_^17^, and even the nature and origin of C_60_ oxidation itself upon exposure to air remains controversial^18^,^19^.

Here we investigate the spectroscopic fingerprint of suspended clusters of C_60_ and (C_59_N)_2_ using near-edge X-ray adsorption fine structure (NEXAFS) spectroscopy and X-ray photoelectron spectroscopy (XPS) combined with a range of density functional theory (DFT) modeling approaches.

Previous near-edge X-ray adsorption fine structure (NEXAFS) spectroscopy studies of fullerenes have relied on thin film samples deposited on surfaces^20^,^21^,^22^. However, during fullerene film preparation, the thermal evaporation step can influence the formation as well as the interaction of the film with the substrate surface, especially in the case of azafullerenes. In addition, surface contamination can have a strong impact on NEXAFS spectra. For this reason, for the NEXAFS analysis, the azafullerene and fullerene powders were sonically dispersed in ethanol, and a drop of each solution was deposited onto lacey carbon films supported on copper transmission electron microscopy (TEM) grids. This allows us to study indirectly supported material located above holes in the lacey carbon film grid, avoiding the interaction of the measured sample region with the substrate.

We model the structures, possible oxidation products and pathways, and simulate spectra of pristine and oxidized fullerene and azafullerene species. Collectively these results allow us to establish a detailed picture of the morphology, electronic structure, and chemistry of these materials, notably including their interaction with water and other oxygen impurities.

## Results and Discussion

### NEXAFS Spectroscopy

Before considering the interaction between oxygen and fullerenes, we first properly characterize the carbon and nitrogen spectroscopic signatures of C_60_ and (C_59_N)_2_.

**Figure 2:**
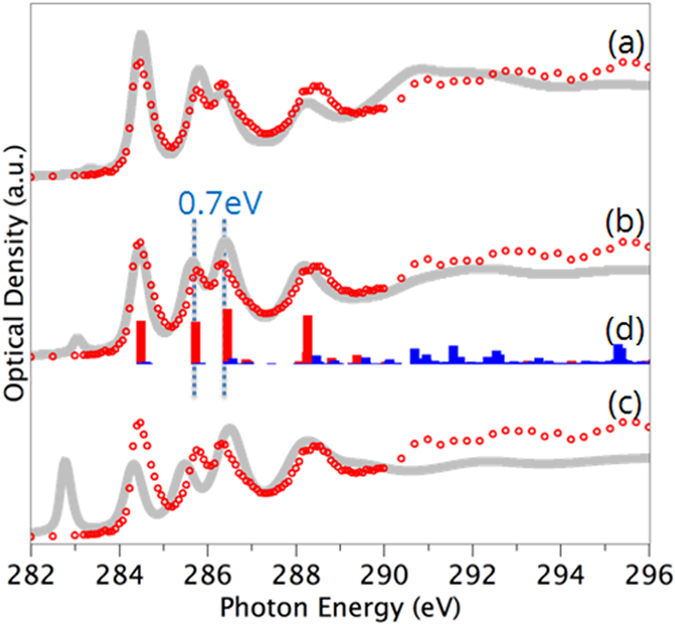
Carbon 1*s* spectrum of C_60_. The experimental scanning transmission X-ray microscopy (STXM) data is plotted in red circles while the calculated spectra are gray lines. Calculations were performed with (**a**) full-core hole (final state), (**b**) half-core hole (Slater transition state) and (**c**) zero-core hole approximation (initial state). (**d**) Breakdown of calculated spectrum into π* (red) and σ* (blue) components.

The experimental and theoretical carbon 1*s* spectra of C_60_ and a projection of the theoretical spectrum onto *p*-components either orthogonal (π*) or tangential (σ*) to the C_60_ cage surface are shown in Fig. 2. The spectrum is characterized by discrete peaks, and in general there is excellent agreement between theory and experiment, both for peak separations and intensities. Our experimental spectrum of C_60_ is very similar to those reported previously in the literature^14^,^21^,^23^,^24^. In order to account for electron-hole interactions we introduced a core-hole in the C 1*s* orbital of the excited carbon atom. Figure 2 shows the effect of removing either 0 (“initial state”), 0.5 (“Slater transition state”^25^) or 1 (“final state”) electron from this 1*s* (C) core orbital (panels a–c, respectively). The best agreement comes from the half core-hole approximation (Fig. 2b), which is used in the rest of our study.

The spectral decomposition in Fig. 2d reveals that the peaks in the low energy range (284–289 eV) have C 1*s* excitation to final states of π* character, while the higher energy spectral features are due to C 1*s* excitation to final states of σ* character. Specific σ* peaks seen in the experimental spectrum from 290–296 eV stand out clearly in the theoretical spectrum decomposition. From this analysis, the first three peaks below 287 eV are assigned to C 1*s* excitations to the four degenerate π* molecular orbitals t_1u_, t_1g_, t_2u_ and a_g_, where the latter two are energetically very close^26^. The last peak at 288.5 eV also corresponds to an a_g_ orbital^27^. We note that the small pre-peak seen in the modeling at around 283 eV is a screening artifact introduced by the half core-hole approximation^28^,^29^ as can be seen by its absense in the full core-hole data. Earlier GGA full core-hole modelling also gave good agreement to experiment^29^, with some variation from our calculated spectra (see Supplementary Information), notably in peak spacings, probably due to differences in basis set construction (the previous study including reduced four-electron effective core potentials for all non-excited carbon atoms, and non-diffusive basis sets for minimization with added diffuse functions for the static exchange Hamiltonian).

**Figure 3:**
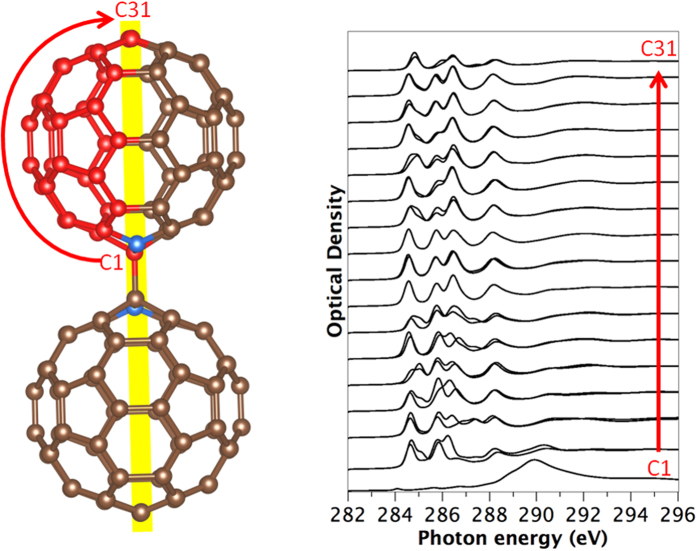
Calculated carbon 1*s* spectra using the Slater transition state, of (C_59_N)_2_ azafullerene for the symmetry inequivalent carbon atoms C_1_ to C_31_. Atom colors: Blue: Nitrogen, Red: inequivalent C atoms, Brown: other carbon atoms.

We next turn to the C 1*s* spectrum of (C_59_N)_2_. The symmetry breaking from nitrogen substitution and fullerene dimerization means there are now 31 symmetrically inequivalent carbon sites, and it is necessary to calculate the spectrum for each of these (Fig. 3). There are 28 inequivalent carbon sites with a multiplicity of four and 3 with multiplicity of two (these lie on the central mirror plane marked in yellow). Of these three, site C1 corresponds to the sp^3^ 4-fold coordinated atoms covalently linking the two fullerene cages together, which is clearly visible in its associated spectrum which lacks the π* related peaks from 284–288 eV.

**Figure 4:**
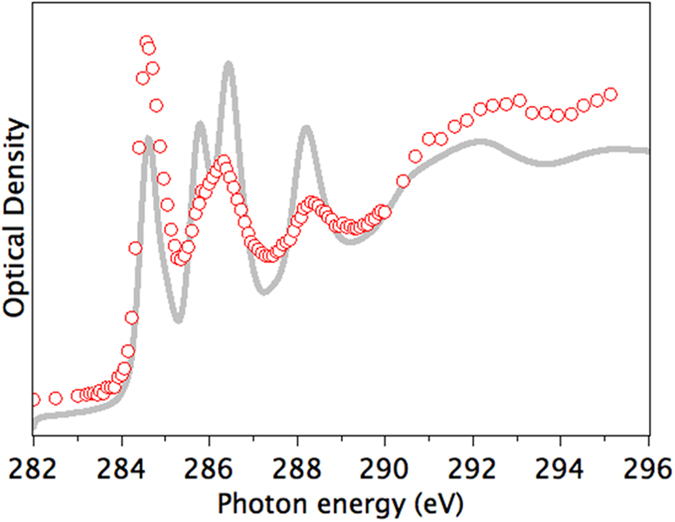
Carbon 1*s* spectrum of (C_59_N)_2_. Experimental STXM data in red circles, calculated spectrum using the Slater transition state in gray is a weighted sum of individual atom spectra shown in Fig. 3.

If we now average these spectra using the appropriate weighting from their multiplicity, we obtain the averaged calculated spectrum, which is shown in Fig. 4 in comparison to the experimental C 1*s* spectrum of (C_59_N)_2_ measured by scanning transmission X-ray microscopy (STXM). Our experimental spectrum of (C_59_N)_2_ is very similar to those reported previously in the literature^14^,^20^,^22^. The experimental C 1*s* spectrum of (C_59_N)_2_ is in general very similar to that of C_60_, apart from additional broadening of the spectral features, notably around 286 eV. These are accurately reproduced by the calculations. This indicates that the perturbation induced by the nitrogen atoms is not very strong, and that the two extra electrons of (C_59_N)_2_ in comparison with C_60_ do not lead to significant occupation of the C-derived low-lying unoccupied states of C_60_.

**Figure 5:**
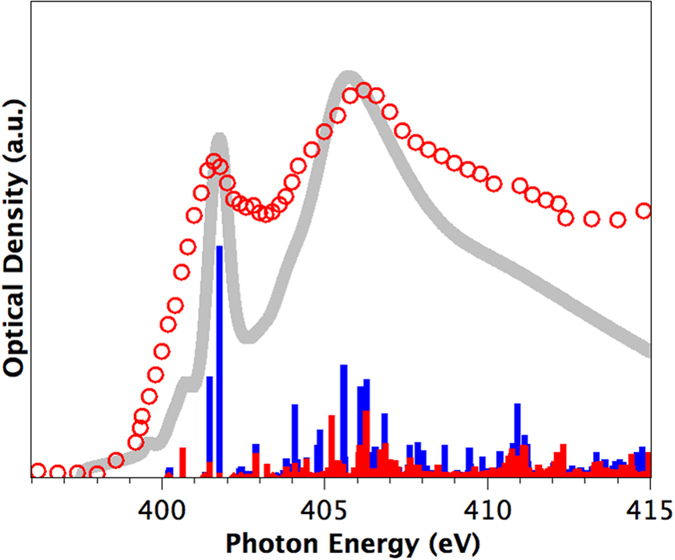
N 1*s* spectra of (C_59_N)_2_ azafullerene. Experimental STXM data in red circles, calculated spectrum using the Slater transition state in gray, projection of calculated spectrum into π* (red) and σ* (blue) components.

The nitrogen 1*s* spectrum of (C_59_N)_2_ is shown in Fig. 5. It exhibits a primary component centered at 401.5 eV and a larger, broader feature at 406 eV consistent with nitrogen neighboring sp^3^-coordinated carbon^30^. When compared to the N 1*s* spectrum of (C_59_N)_2_ reported by Schulte *et al*.^14^ there is considerable disagreement, in particular the separation of the two main peaks is different (4.8 eV in this work, 5.2 eV in ref. 14) and there is a major difference in the width and relative intensity of the first peak which can not be explained by instrumental differences. In particular the N 1*s* spectrum of (C_59_N)_2_ reported by Schulte *et al*.^14^ has a rather sharp peak at 401.5 eV (fwhm ~1 eV), whereas the corresponding feature on our spectrum is much broader (fwhm ~2 eV). We explored the possibility the signal in the Schulte *et al*. spectrum^14^ could be from trapped gaseous N_2_. Subtraction of N_2_ gas signal to remove this sharp peak does result in a much broader first peak, closer to that we have measured, but there is still a poor match to our result.

By contrast good agreement is found between our measured and calculated N 1*s* spectra, notably in the features in the extended σ* states and the low energy shoulder to the 401.5 eV peak. We note that our theoretical and experimental data correctly reproduces the key spectral features found in a recent simulated (C_59_N)_2_ NEXAFS N 1*s* spectrum^31^, including primary peak spacings and the presence of the small pre-peak shoulder. This gives us additional confidence in our results

A key difference between the N 1*s* and C 1*s* spectra is that while the primary C 1*s* peak has strong π* character, the primary N 1*s* peak is mainly σ* in character. This is consistent with previous findings for EELS spectroscopy of nitrogen doping in single-walled carbon nanotubes^28^. In the current study we can see however that some π* character is responsible for the low energy shoulder of this peak, due to slight deviation from full tetrahedral configuration imposed by the fullerene cage environment.

The spectroscopic influence of nitrogen substitution in the fullerene cage is twofold: first, the stronger core potential offered by the nitrogen atom manifests itself in a low-binding energy shoulder split-off from the highest occupied molecular orbital (HOMO), carrying significant nitrogen character^32^. This is, however, not to be associated with simple filling of the lowest unoccupied molecular orbital (LUMO) of the C_60_, as the excess electronic charge stays relatively localized on the nitrogen sites. The substitutional nitrogen atom in C_59_N is sp^3^-bonded, i.e. forms three σ bonds with its carbon neighbors, with its remaining two valence electrons forming a stable lone pair. This means the loss of a π bond in the associated hexagon-hexagon double bond, creating a neighboring carbon radical with an unpaired p_z_-electron. The carbon radicals of neighboring cages combine to form a σ bond between them. Thus, in terms of bonding, the conversion from C_60_ to (C_59_N)_2_ involves the elimination of only one π bond per fullerene, and some structural modification of nearby σ bonds around the substitutional and cross-linking sites (Supplementary Figure 3).

The heteroatom introduces changes to the electronic structure of the fullerene cage, observed as spectral broadening and the appearance of two structures in between the three highest valence band peaks in photoemission^32^. As compared to the energy gap of C_60_ (∼1.86 eV), the energy gap between occupied and unoccupied states is smaller in (C_59_N)_2_ at 1.4 eV (ref. 22).

To summarise this section, we find good agreement between theory and experiment for the C 1*s* and N 1*s* spectra of C_60_ and (C_59_N)_2_. Small differences in peak positions and intensity are observed between these two species, caused by the relatively localised disruption to the fullerene bonding in the azafullerene dimer. While the first peak in the C 1*s* spectrum has π* character, in the N 1*s* spectrum this peak has primarily σ* character with a small low energy π* shoulder. It should be noted that the use of a statically screened electron-hole interaction via the Slater transition state allows accurate reproduction of the present NEXAFS data for both C_60_ and (C_59_N)_2_. Additionally, such an approach means that for the carbon 1*s* spectrum for (C_59_N)_2_, a weighted spectrum based on 31 half core-hole spectra for each inequivalent carbon site can be determined, allowing analysis of the contribution of each carbon atom to the resulting experimental spectrum. This demonstrates that while the static approach does not allow an explicit treatment of core-hole interactions, it is nonetheless a very powerful tool for the analysis of complex spectra.

### X-Ray Photoelectron Spectroscopy

The discussion thus far has concerned the quantitative agreement between modeling and spectroscopy for C 1*s* and N 1*s* NEXAFS spectra. We next compare calculated C 1*s* and N 1*s* core level binding energies to those measured with XPS. An absolute energy comparison is essential to understand the interactions between molecules and (aza)fullerenes.

We find that although carbon atoms near the nitrogen site(s) do exhibit different calculated C 1*s* energies than those from pristine “bulk” C_60_, the binding energies are less than 40 meV higher already for next-nearest neighbors away from the N. Thus while the experimental C 1*s* spectrum is in theory a convolution of several peaks, within the experimental accuracy it is well described by the bulk value. For the dimer, we obtained N 1*s*–C 1*s* energy separations of 115.65 eV (ΔSCF) and 115.73 eV (ΔKS), in excellent agreement to our experimentally measured separation of 115.5 eV. This demonstrates the strength of this approach for the quantitative determination of relative core-level energies, with the ΔSCF method featuring the additional benefit of having the same atomic reference energies for both the N 1*s* and C 1*s* calculations.

We note further that although it is not experimentally accessible due to dimerization, the calculated N 1*s*–C 1*s* energy separation for the single C_59_N radical is 116.10 eV, implying that the interaction in the dimer lowers the N 1*s* level by about 0.4 eV (with the Kohn-Sham eigenvalues approximating the initial state energies showing a slightly larger difference of 0.7 eV). This is presumably due to slight differences in the chemical environment of the N atom and core-hole screening in the two systems.

### Calculated Interaction with Atomic Oxygen Species

**Figure 6:**
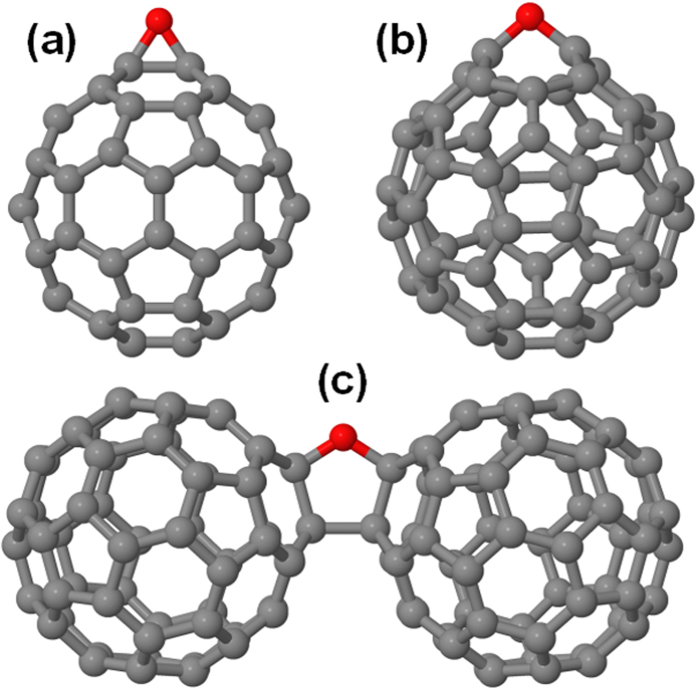
Stable oxygen-fullerene complexes: C_60_O with oxygen in the (**a**) epoxide and (**b**) annulene configurations (**c**) ether-oxygen-bridged fullerene dimer C_60_-O-C_60_. Carbon atoms are shown in grey, oxygen in red.

We next turn our attention to the nature of oxygen contamination in fullerene and azafullerene samples, which remains an open question in the literature. Both epoxide and annulene fullerene structures (Fig. 6a,b) have been successfully synthesized^33^,^34^. Density functional theory (DFT) studies of single oxygen addition to heterofullerenes found that C_59_N and C_59_HN showed similar epoxide formation and binding energies as C_60_ (ref. 17). However, it is not obvious how epoxides and annulenes would be produced via spontaneous O_2_ incorporation in C_60_ crystals upon air exposure. The most stable configuration for a single oxygen atom is cross-linking a fullerene dimer^35^,^36^,^37^,^38^ via a 2 + 2 cycloaddition reaction combining double bonds in neighboring C_60_, where the oxygen atom adopts an ether configuration in one of the cross-linked bonds (Fig. 6c). Dry air studies have suggested the presence of interstitial oxygen molecules sitting in the octahedral voids of the crystal^18^, and modeling has confirmed the octahedral site as the most stable for O_2_ incorporation^38^.

**Figure 7:**
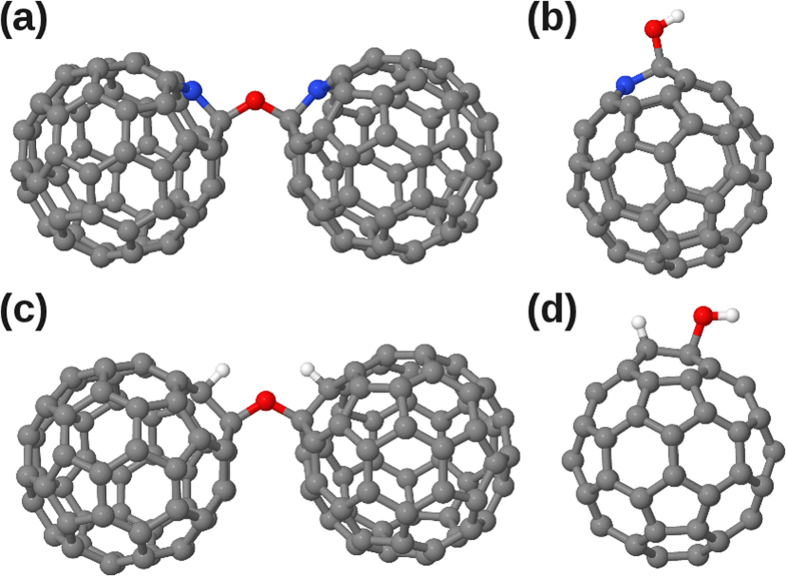
Structures of (**a**) C_59_N-O-C_59_N (**b**) C_59_N(OH), (**c**) C_6O_H-O-C_60_H and (**d**) C_60_H(OH). Carbon atoms are shown in grey, oxygen in red, nitrogen in blue and hydrogen in white.

The most stable configuration that we found for a single oxygen atom in (C_59_N)_2_ structures is the analogue of the bridging configuration of C_60_ dimers, i.e. an oxygen atom in an ether configuration in the cross-linking bond between (C_59_N) pairs in the dimer (Fig. 7a). As compared to C_60_-O-C_60_ (Fig. 6c), replacing the sp^3^-cross-linked carbon atoms with two non-bonded nitrogen atoms removes the second cross-linking bond between the fullerene cages and relieves significant strain.

### Calculated Interaction with Water

FTIR spectra have demonstrated the presence of both O_2_ and H_2_O in C_60_ crystals exposed to air^19^, and testing C_60_ in air with varying humidity has suggested that the observed O 1*s* XPS signal was caused by H_2_O exposure^39^. Indeed, C_60_ has a high affinity for water, as shown by its high free energy of hydration (−90.5 mJ/m^2^)^40^.

Before analyzing our experimental data, we first explore theoretically the feasibility of molecular H_2_O interaction with fullerenes and azafullerenes. To the best of our knowledge, there is little modeling of the H_2_O-C_60_ interaction. Tsetseris *et al*. showed that C_60_H(OH), i.e. H_2_O dissociated to saturate a C_60_ double bond, is 0.1 eV less stable than H_2_O in an octahedral void in the C_60_ crystal^38^, and models have been proposed of hydration cages forming around C_60_ when in solution^41^,^42^,^43^.

We calculate the binding energy of H_2_O to isolated “gas-phase” C_60_ to be 0.34 eV, increasing to 0.56 eV when the H_2_O molecule is placed in the octahedral interstitial void in fcc C_60_. The binding energy of H_2_O to gas phase (C_59_N)_2_ is 0.32 eV, very similar to that for H_2_O binding to C_60_. Since the nitrogen atoms are close to the cross-linked carbon atoms, they are spatially inaccessible to the H_2_O molecule, which is forced to sit further away on the cage surface and does not interact strongly with the nitrogen. The result again reflects the earlier finding that the perturbation of the fullerene cage caused by nitrogen substitution remains localized around the nitrogen and its neighbors, with the rest of the cage closely resembling pristine C_60_.

Considering potential reactions of (C_59_N)_2_ with water, one possibility is that H_2_O could saturate the C-C cross-linking bond between the cages, creating a C_59_NH/C_59_N(OH) pair. However, we found this structure to be 0.27 eV less stable than C_59_N-C_59_N with a neighboring H_2_O molecule. Additionally, we calculate an extremely high reaction barrier of 3.94 eV for this process, due to the difficulty in forcing the H_2_O molecule between the two fullerene cages in close proximity. We hence conclude that water is likely to remain molecular in interstitial voids if present within the bulk azafullerene sample.

Considering now possible reactions involving H_2_O and C_60_, a new structural analogue to C_59_N-O-C_59_N can be proposed, namely C_60_H-O-C_60_H (Fig. 7c). This is 0.13 eV more stable than H_2_O sitting in the interstitial space between two C_60_ molecules (which we find isoenergetic with C_60_ neighboring C_60_H(OH) (Fig. 7d), close to the result of Tsetseris *et al*.^38^). C_60_H-O-C_60_H thus represents the lowest total energy structure we obtained with a single H_2_O molecule and C_60_. However, entropic considerations will not favor this structure at finite temperatures, and similarly to the azafullerene case, there is a significant reaction barrier of 2.40 eV that needs to be overcome to form this structure from H_2_O + 2C_60_. It is possible such a process could be catalyzed, either by doping (in a -1 charge state this barrier drops to 1.66 eV with C_60_H-O-C_60_H now 0.24 eV more stable) or photocatalyzed (in the triplet state the calculated barrier drops to 1.67 eV).

Thus, even if catalyzed, the reaction barriers remain high enough that under conventional room temperature conditions we expect H_2_O in the presence of C_60_ crystals to remain in molecular form, as in azafullerenes.

### Oxygen X-Ray Photoelectron Spectroscopy

**Figure 8:**
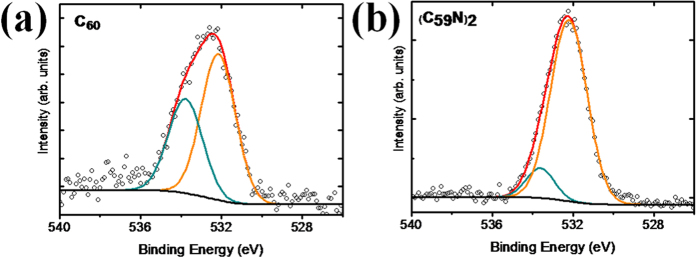
O 1*s* XPS signal for (**a**) C_60_ and (**b**) (C_59_N)_2_ powders.

We next turn to our experimental oxygen data and its interpretation. Figure 8 shows the O 1*s* XPS signal of our fullerene and azafullerene samples (with the C 1*s* found at 284.6 eV). While both samples show a high intensity component at 532.2 eV, the C_60_ sample also shows another prominent oxygen component at 533.8 eV, only weakly present in the azafullerene sample despite the overall oxygen concentration being slightly higher there. In general, the oxygen concentration is comparable with previous studies^39^. The elemental compositions of the samples are shown in Table 1.

The spatial resolution of STXM means we are able to collect NEXAFS spectra exclusively from single, indirectly supported crystalline regions. Notably we can compare our NEXAFS measurements, which are dominated by the bulk response of the crystallites, with the surface-dominated XPS data. In the individual C_60_ crystallites we did not detect any O 1*s* signal with NEXAFS. The azafullerene sample, which is more porous and less crystalline, did show a weak O 1*s* NEXAFS signal; however, comparison with theoretical modeling suggests this is due to ethanol contamination (see Supplementary Materials), which was used as a dispersant for the NEXAFS sample preparation.

The STXM-NEXAFS result therefore suggests that the observed O 1*s* XPS signal does not come from the bulk of the fullerene and azafullerene materials. There are thus two possible explanations for the signal. The first is that it comes from amorphous regions present elsewhere in the sample. The second is that it comes from surface/sub-surface adsorbed species, to which XPS is particularly sensitive, but which would be masked by the NEXAFS signal of the bulk. It is important to note that there can be no XPS signal corresponding to ethanol, since solvent was not used to make the XPS samples.

In order to assign the two peaks at 532.2 and 533.8 eV identified by XPS, we calculated the O 1*s* energies for all the structures shown in Figs 6 and 7 using the ΔSCF method (the ΔKS method yielded slightly lower binding energies for both the C 1*s* and O 1*s* levels, but their separation was almost identical between the two methods in all cases), with the results given in Table 2. Considering first the experimental O 1*s* XPS peak at 532.2 eV, the energy given in Table 2 for the OH group in C_60_H(OH) is close to this value at 532.53 eV. However, the calculated O 1*s* binding energies of ether-bound oxygen in C_59_N-O-C_59_N and C_60_H-O-C_60_H are also very similar; hence, we cannot conclusively assign the measured energies to one specific bonding, but suggest it corresponds to either hydroxyl- or ether-bonded oxygen in the sample.

The second experimental peak at 533.8 eV has a higher binding energy than any of our calculated structures in Table 2, and indeed, such a high value is strongly indicative of water. Onoe *et al*. measured XPS for C_60_ crystals exposed to humid air^39^, and found an O 1*s* peak at 533.5 eV, in good agreement with our value. They attributed this to water bound on the surfaces of the fullerene crystals. Given the ultra-high vacuum (10^−9^ mbar) in our XPS apparatus, we can safely assume that gas-phase H_2_O is unlikely (and would have an even higher O 1*s* of 535.75 eV)^44^. In other studies of water on metal and oxide surfaces, a downshift for surface adsorbed H_2_O to as low a value as 532.8 eV has been reported^44^, but screening on such surfaces is likely stronger than in the fullerene crystal. Since NEXAFS has excluded the presence of significant amounts of H_2_O within the crystal, the bound H_2_O observed in XPS must necessarily be near the crystal surface (the estimated maximum escape depth of our photoelectrons is ~2–3 nm). The lower intensity of the 533.8 eV O 1*s* signal in the C_59_N sample, arising from water, is also consistent with its decreased crystallinity as compared to C_60_ (see Supplementary Materials).

To directly simulate the core level binding energy of bound water, we created a model of eight C_60_ molecules taken from the infinite FCC lattice containing the relevant pore, surrounded by 15 Å of vacuum on all sides. Then, we ran two core level calculations using the ΔKS method: one with a H_2_O molecule in the pore, and another with the H_2_O placed at the corner of the simulation cell, far from the fullerenes. Thus the contents of the cell were the same in both cases (483 atoms with a total of 1928 valence electrons), and any error in screening our method makes for molecules is likely of similar magnitude. We should note that these are the largest core hole calculations ever reported to the best of our knowledge.

After correcting the resulting O 1*s* values so that both C 1*s* levels again match the experimental value, we found adsorption of H_2_O into the pore to downshift its O 1*s* level by 1.0 eV. Although this is not quite as much as the 2 eV difference between our higher BE component (533.8 eV) and a reference value for gas-phase H_2_O (535.75 eV), it is of similar magnitude and in the correct direction. Additional calculations for a hydration shell^41^ of 80 H_2_O molecules around C_60_ gave nearly the same O 1*s* binding energy for H_2_O at the C_60_ surface as for the single H_2_O molecule in the C_60_ FCC crystal pore. Thus the calculations are consistent with the assignment of this peak to surface or sub-surface water.

The O 1*s* XPS results strongly resemble findings for surface bound water on metals, where two O 1*s* peaks in XPS are commonly observed (for a review see ref. 45), and assigned to H_2_O and hydroxyl groups in the surface water layer, respectively. The presence of bound hydroxyl groups enhances water binding^46^. For example, two XPS peaks at 530.8 and 532.4 eV are observed for Cu(110) cleaned in UHV and then exposed to water at room temperature^46^, similarly, water on Ru{0001} shows two O 1*s* XPS peaks at 531.0–530.8 eV and 532.7–532.3 eV (the precise position varies with coverage)^47^. There is also precedent for water layer formation on carbon nanomaterials; for example, bilayer water formation has been observed on the surface of graphene under UHV conditions^48^. Thus our calculations, the XPS data, and comparison to the literature strongly supports the interpretation that our higher binding energy component is due to a surface water layer, resistant to evaporation even in the spectroscope vacuum, with the lower binding energy component due to hydroxyl groups present in this layer. We note as further evidence of the strong layer binding that when we applied an annealing step at 70 °C within the XPS chamber prior to measurement, the resultant O 1*s* signal remained unchanged.

In our case there was no deliberate introduction of water to the samples, which was rather adsorbed by the samples from the humid air of the laboratory over time. Daylight exposure in the laboratory can also help explain the presence of the strong hydroxyl signal, since it has been demonstrated that exposure of C_60_ to UV light in the presence of water leads to hydroxyl radical creation^49^.

## Discussion and Conclusions

Isolated suspended clusters of C_60_ and (C_59_N)_2_ were analyzed by a combination of nanoscale NEXAFS, XPS and various DFT approaches. Our study clearly shows the benefit of combining different complementary theoretical and experimental techniques in order to characterize such complex samples as completely as possible.

We show that the extra electron in the azafullerene is predominantly localized on the N atom, and interpret the primary low energy peaks in the C 1*s* as coming from carbon π* states, whereas the primary low energy peak in the N 1*s* are from σ* states. Interpretation of the observed oxygen signal is complicated, but we propose that NEXAFS identifies the presence of ethanol solvent within the azafullerene crystals. In contrast, as more surface-sensitive, the XPS characterization identifies the presence of a surface water layer containing hydroxyl groups on both samples, with lower concentrations in the azafullerene sample. The water must necessarily be rather strongly bound given the ultra-high vacuum conditions used for XPS, which is corroborated by the large O 1*s* downshift of around 2 eV as compared to gas-phase water. This wetting behaviour strongly resembles that observed on many metal surfaces.

The presence of high levels of surface/subsurface adsorbed water in fullerene crystals, even under ultra-high vacuum conditions, has many important implications. One in particular is in the field of astrochemistry and astrobiology. Recent studies from the Spitzer radio telescope have identified large amounts of crystalline C_60_ in interstellar outflows^3^. We propose here that the C_60_ crystal surfaces may serve as substrates facilitating the formation of amino acids in the interstellar medium, and ultimately the earliest stages of life.

If water is strongly surface bound to C_60_, we could speculate that this may also be the case for other gaseous species. Surface reactions with water could then be catalyzed by kinetic confinement on the two-dimensional C_60_ crystal surface, with further mechanical confinement brought about by the surface rugosity. Surface reactions could be additionally catalyzed by UV-adsorption and energy transfer from the underlying fullerene crystal. C_60_ is known to be a highly efficient UV-absorber that promotes radical formation, for example converting triplet O_2_ to the singlet state with very high quantum yield^50^, and can in the presence of water and UV generate hydroxyl radicals^49^. Thus C_60_ crystals would be able to absorb the large amounts of available high energy UV-light present in and around such interstellar outflows, and direct it towards catalyzing surface reactions.

## Methods

C_60_ was purchased from Bucky USA (99.5% grade), stored in the lab for several months and used as received. The details of azafullerene (C_59_N)_2_ synthesis have been reported elsewhere^51^. Briefly, starting from C_60_, [60]-N-MEM ketolactam is isolated following a two-step procedure. A degassed solution of the ketolactam in o-dichlorobenzene is refluxed under argon for 15 min with an excess of p-toluenesulfonic acid. After the reaction mixture is cooled, flash column chromatography using silica gel with toluene as eluent afforded azafullerene (C_59_N)_2_ as the least polar fraction. The material was further purified by high-performance liquid chromatography (Buckyprep column 20 × 250 mm, toluene eluent, 20 mL/min flow rate, 333 nm UV detection). The final HPLC trace shows only a single peak after recycling, the profile indicating the purity is >99.99%. The azafullerene samples after purification by HPLC, were stored in flat clear vials (4 mL/14.75 × 45 mm), with screw neck and with clear screw cap closed top Teflon, 1.5 mm (purchased from MACHEREY-NAGEL GmbH & Co. KG) at 25 °C and humidity 20%. They were prepared and transported between synthesis and characterization laboratories over a period of a few months. Storage in characterization laboratories was far from other chemical products.

STXM spectromicroscopy was used to analyze selected regions of the sample, taking advantage of its nanoscale spatial resolution^52^,^53^. The carbon 1*s* spectra were recorded using the ambient STXM on the 10ID-1 soft X-ray spectromicroscopy (SM) beamline at the Canadian Light Source (CLS) in Saskatoon, Canada. This beamline has an energy resolving power E/ΔE > 5000^54^. In STXM monochromatic X-rays of different energies are focused onto the sample by a Fresnel zone plate^55^ and the transmitted X-rays detected. The spectra were obtained by (x, y) raster-scanning the sample at the focus of the X-rays over a range of photon energies so that a sequence of images at different energies was acquired, known as a “stack”^56^. The nitrogen 1*s* spectra were recorded on a different region of the same sample with the STXM on beamline 5.3.2.2 at the Advanced Light Source in Berkeley, USA. Data processing was similar in both cases. The oxygen 1*s* spectra were measured with the full field transmission X-ray microscope (TXM) at the U41-FSGM beamline, BESSY II electron storage ring, Berlin, Germany^57^. The spectra were recorded at room temperature in transmission mode by taking a sequence of images over a range of photon energies covering the investigated adsorption edges with a spectral resolution larger than 5000. The zone plate used provided a spatial resolution of 25 nm. Stack alignment, conversion to optical density, and extraction of NEXAFS spectra from small, free-standing regions were performed using the aXis2000 software^58^. Since the photon flux at the sample varies as a function of energy and time, the aligned image sequences were converted to optical densities by dividing the transmitted signal recorded on the sample region by the incident photon flux recorded in a sample-free region. Thus, the ultimately acquired image sequences at different energies provide spatially resolved NEXAFS spectra.

X-ray photoelectron spectroscopy (XPS) measurements were performed in a VG Escalab 220i XL with background pressure of 1×10^−10^ Torr, equipped with a source of monochromatic Al Kα X-rays (hν = 1486 eV). The escape depth of the photoelectrons is typically 2–3 nm, and the energy resolution was 0.6 eV. An electron gun (~1 eV) was used to compensate built-up charge on the specimen surface during the measurements. The samples for the XPS measurements were prepared by pressing the specimen into a pellet, avoiding the need for a solvent. A conductive double-face tape was used to attach the pellet to a sample holder.

Density functional theory calculations of different fullerene and azafullerene structures, their complexes with oxygen and water, and associated formation barriers were performed using the LDA-PW91 exchange-correlation functional^59^ as implemented in the AIMPRO code^60^,^61^,^62^. Spin-polarized calculations were carried out using supercells, fitting the charge density to plane waves with an energy cutoff of 150 Hartrees. Relativistic pseudopotentials generated by Hartwigsen, Goedecker and Hutter were used^63^. 28, 40 and 40 independent Gaussian functions were used as basis sets for carbon, nitrogen and oxygen respectively. Periodic boundary conditions were applied, with supercell sizes checked and chosen to be sufficiently large (vacuum distance between all structures being larger than 13 Å) to avoid interactions between fullerenes in neighboring cells. Electronic level occupation was obtained using a Fermi occupation function with kT = 0.04 eV. Absolute energies were converged in the self-consistency cycle to better than 10^−8^ Ha. Atomic positions were geometrically optimized until the maximum atomic position change in a given iteration dropped below 10^−5^ Bohr radii. Diffusion barriers were calculated using the climbing nudged elastic band method, with typically 20 images between the initial and final structure.

Simulated X-ray adsorption spectra were calculated using the WIEN2k program package^64^ with the LDA functional and using the GGA-PBE optimised atomic structures. The GGA functional is better suited for the geometry optimisation, whilst our test calculations demonstrated that LDA gave slightly better C 1*s* peak seperations for C_60_ than GGA for NEXAFS simulations (see Supporting Materials). In order to describe properly the impact of the two-particle electron-hole interactions on the XAS spectra, one should go beyond DFT by using the Bethe-Salpeter approach^65^ or time-dependent DFT^66^. Here, due to the size of the systems, we have chosen to mimic these many-body effects by considering a static screened electron-hole interaction using the Slater transition state, i.e. a half-reduced occupation in the C 1*s* and N 1*s* core levels when calculating the the C and N 1*s* NEXAFS of C_60_ and (C_59_N)_2_. A half-electron uniform background was added to perform these calculations. The broadening of our theoretical spectra was done in two steps by first considering a Lorentzian to account for the core-hole lifetime using tabulated values^67^, and adding an energy-dependent broadening to account for the excited state lifetimes^68^. For the energy-dependent broadening we considered a plasmon frequency of 8 eV for both C 1*s* and N 1*s* spectra. In order to compare our theoretical spectra with experiment, an energy shift which depends on the system and level of theory is needed. For the three C 1*s* spectra of C_60_ using 0, 0.5 and 1 core-holes, energy shifts of 282.7, 283.0 and 283.3 eV were used, respectively. For (C_59_N)_2_ energy shifts of 284.0 and 399.5 eV were used for the N 1*s* and C 1*s* spectra, respectively.

For calculations of the C, N and O 1*s* core-level binding energies, we utilized two methods based on density functional theory total energy differences, as implemented in the grid-based projector-augmented waves (PAW) simulation package GPAW^69^,^70^: a standard delta Kohn–Sham (ΔKS) calculation with a projector-augmented wave dataset including an explicit core-hole on an atom of interest^71^, and a novel all-electron extension of the delta self-consistent field (ΔSCF) method^72^. In the latter, the core electrons are included in the valence, enabling an explicit all-electron calculation within the PAW scheme^73^. Exchange and correlation for these calculations were estimated by the Perdew-Burke-Ernzerhof (PBE) generalized gradient approximation^74^, yielding significantly more accurate core level binding energies than the LDA^72^. In each case, we calculated via total energy differences the respective 1*s* energy and compared this to the C 1*s* energy for a carbon atom far away from the N site(s).

## Additional Information

**How to cite this article**: Erbahar, D. *et al*. Spectromicroscopy of C_60_ and azafullerene C_59_N: Identifying surface adsorbed water. *Sci. Rep.* **6**, 35605; doi: 10.1038/srep35605 (2016).

## Supplementary Material

Supplementary Information

## Figures and Tables

**Figure 1:**
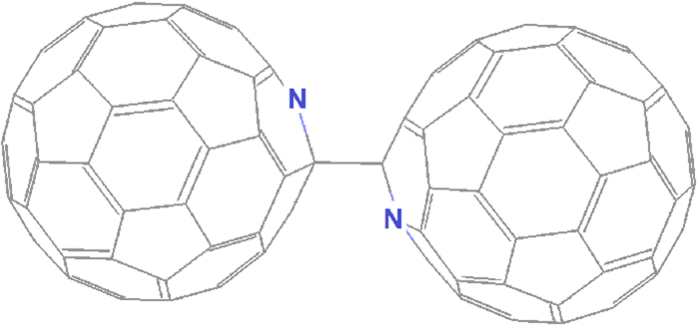
Structure of the azafullerene dimer (C_59_N)_2_.

**Table 1:** Elemental compositions (±0.5%) determined by XPS for (a) C_60_ and (b) (C_59_N)_2_ powders.

Sample	C	N	O	O (532.2 eV)	O (533.8 eV)
C_60_	97.0%	—	3.0%	1.8%	1.2%
(C_59_N)_2_	94.5%	1.5%	4.0%	3.5%	0.5%

**Table 2:** Calculated O 1*s* core-level binding energies for the different structures in Figs 6 and 7.

Structure	Calculated O 1*s* [correction] (eV)
C_60_H-O-C_60_H	532.56 [+0.39]
C_60_-O-C_60_	532.69 [+0.39]
C_60_-O- (annulene)	533.43 [+0.39]
C_60_ > O (epoxide)	533.00 [+0.39]
C_60_H(OH)	532.53 [+0.39]
C_59_N(OH)	533.01 [+0.26]
C_59_N-O-C_59_N	532.49 [+0.26]

The energies are shifted so that the C 1*s* energy of the corresponding unmodified structure (C_60_ or (C_59_N)_2_) matches the experimental value of 284.6 eV, with the applied corrections given in brackets.
