# Supplementary material for: Spectromicroscopy of C60 and azafullerene C59N: Identifying surface adsorbed water
Source: Sci Rep. 2016 Oct 17;6:35605. doi: 10.1038/srep35605 (PMC5066267; doi:10.1038/srep35605)
Supplement: Supplementary Information [file srep35605-s1.doc]

Supplementary Materials

**Spectromicroscopy of C_60_ and azafullerene C_59_N: Identifying surface adsorbed water**

Dogan Erbahar,^1,2^ Toma Susi,^3^ Xavier Rocquefelte,^1,4^ Carla Bittencourt,^5^ Mattia Scardamaglia,^5^ Peter Blaha,^6^ Peter Guttmann,^7^ Georgios Rotas, ^8^ Nikos Tagmatarchis,^8^ Xiaohui Zhu,^9^ Adam P. Hitchcock,^9^ and Chris P. Ewels^1,*^

^1^ Institut des Matériaux Jean Rouxel, Université de Nantes, CNRS, Nantes, France.

^2^ Physics Department, Gebze Technical University, Gebze, Turkey.

^3^ University of Vienna, Faculty of Physics, Boltzmanngasse 5, A-1090 Vienna, Austria.

^4^ Institut des Sciences Chimiques de Rennes, UMR 6226 CNRS, Université de Rennes 1, Rennes, France

^5^ Chemistry of Interaction Plasma-Surface (ChIPS), University of Mons, Mons, Belgium.

^6^ Institute for Materials Chemistry, TU Vienna, A-1060 Vienna, Austria.

^7^ Helmholtz-Zentrum Berlin für Materialien und Energie GmbH, Institute for Soft Matter and Functional Materials, Berlin, Germany.

^8^ Theoretical and Physical Chemistry Institute, National Hellenic Research Foundation, 48 Vassileos Constantinou Avenue, 11635 Athens, Greece.

^9^ Dept. of Chemistry and Chemical Biology, McMaster University, Hamilton, ON, L8S 4M1, Canada.


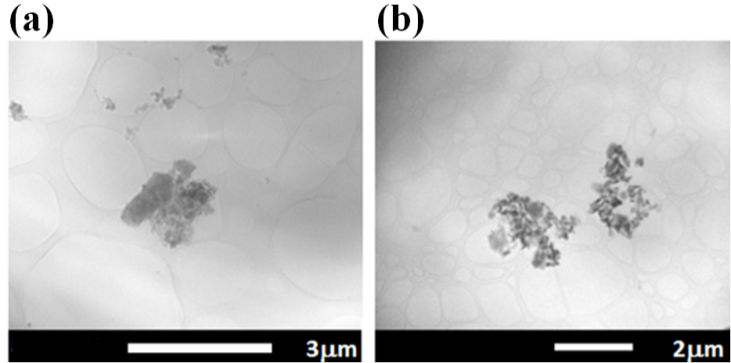


Supplementary Figure 1 Typical transmission X-ray microscopy images of the azafullerene sample. The images were recorded at a photon energy of 525.0 eV. Each pixel is 10 nm and has an associated NEXAFS spectrum. a) “Solid” particles (arrow) and b) packed crystallites.

While C_60_ crystals are face-centred cubic packed above -20° C (below this the overall symmetry is maintained but fullerene rotation ceases, breaking the symmetry locally), (C_59_N)_2_ forms hexagonal close packed crystals.^1^ From our DFT calculations we obtain a lattice C_60_ constant of 14.02 Å, in good agreement with reported experimental results (14.11Å).^2^ Supplementary Figure 1 shows a typical transmission X-ray microscopy (TXM) image of the azafullerene sample on a lacey carbon film. This confirms earlier transmission electron microscopy studies^1^ which identified two distinct sample areas. The first are “solid” particles where diffraction demonstrates the material is non-crystalline, with the local structure explained as forming through the rapid degassing and associated solvent loss creating porous amorphous spheres. The second morphology is collections of much smaller crystallites packed in a hexagonal lattice (*P63/mmc, a* = 9.97 Å and *c* = 16.18 Å).^1^ Reference 10 in the main paper provides further XRD studies of this material.

**STRUCTURAL COMPARISON BETWEEN C_60_ AND AZAFULLERENES**


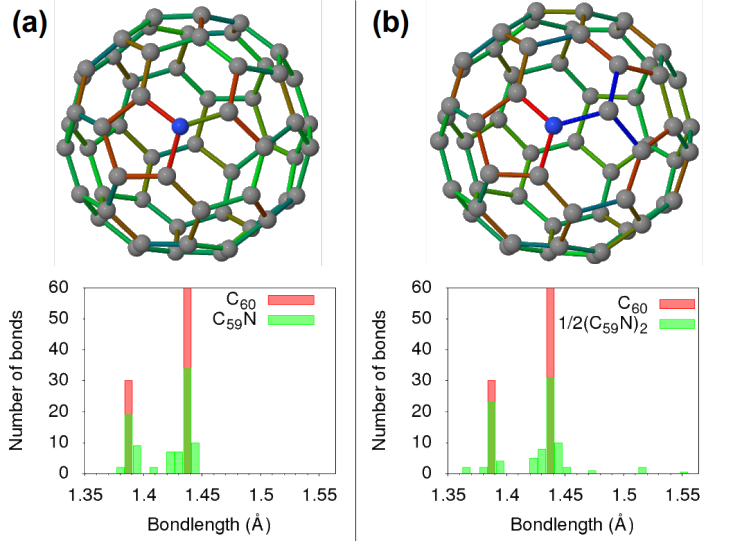


**Supplementary Figure 2** (a) C_59_N and (b) (C_59_N)_2_ with bond lengths coloured to indicate compression (red), no change (green) or dilation (blue) as compared to pristine C_60_. Only one half of (C_59_N)_2_ shown for clarity. Histograms of bond length distributions are shown below the structures, demonstrating a larger spread in σ than π bonds after the incorporation of N in the cages. Carbon atoms are shown in grey, nitrogen atom in blue.

The localization of the additional N valence electrons and the associated neighboring carbon radical is reflected in the calculated DFT structure. Supplementary Figure 2 shows the change in bond lengths as compared to pristine C_60_, for both the C_59_N monomer and the (C_59_N)_2_ dimer (only one of the dimer fullerene cages is shown for simplicity). The bonds are colored based on their change in length compared to ideal C_60_ bonds (1.386 Å and 1.439 Å). Unchanged bonds are green, shorter bonds are red, and longer bonds blue, with the rgb values scaled as (Δbondlength)^0.3^ to make small bond length changes more visible. The major variations are strongly localized around the substitutional site in the dimer, while the rest of the cage is largely unaffected. Notably it can be seen in the histogram of the distribution of bond lengths in (C_59_N)_2_ as compared to C_60_ that there is more variation in the single (σ) bonds than in the double (π) bonds. This is consistent with the measured spectra, where the first C 1*s* → π* peak remains virtually unchanged between C_60_ and (C_59_N)_2_, while there are more changes in the higher energy C 1*s* → σ* fine structure.


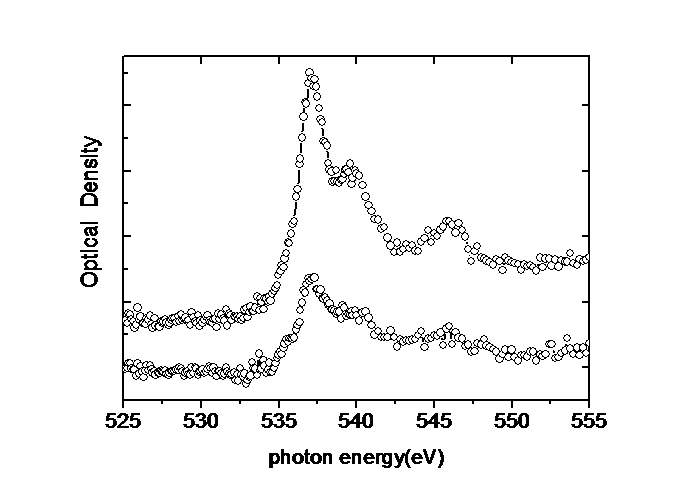


Supplementary Figure 3 NEXAFS signal of the O K-edge measured with TXM at two positions of the spherical-like particle region of the azafullerene sample (Supplementary Figure 1b).

**OXYGEN NEXAFS SIGNAL IN AZAFULLERENES**

The O K-edge from the azafullerene sample for two different sample positions is shown in Supplementary Figure 3. The shape of the response does not correspond to the spectrum of water in either vapor, liquid or solid phases,^3-5^ although bonding in the octahedral voids could conceivably somewhat alter the response. However, the spectrum is quite similar to the response of methanol,^6^ suggesting the presence of C-OH bonding as its origin.

Since the NEXAFS samples were prepared from an ethanol solution, this seems the likely source of the hydroxyl signal observed in this case for the azafullerene regions. Observation of solvent only in the azafullerene sample would be reasonable, given its higher porosity and lower crystallinity than the pure C_60_.

**GGA vs LDA FOR C60 NEXAFS CALCULATIONS OF C1S FOR C60**


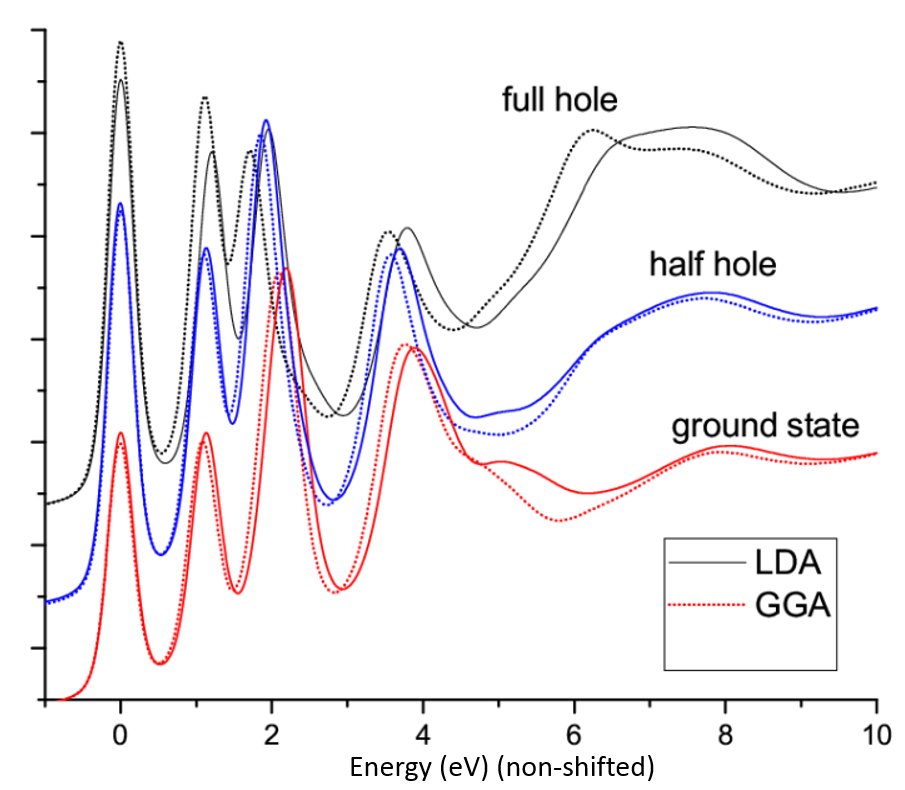


**EXPERIMENTAL XPS FOR C1s AND N1s IN (C_59_N)_2_ SAMPLES**

**REFERENCES**

1 Prassides, K. *et al.* Spheres of Spheres of Azafullerene in the Solid State. *Chem Mater* **8**, 2405-2408, doi:10.1021/cm960282v (1996).

2 Heiney, P. A. *et al.* Orientational ordering transition in solid C60. *Phys Rev Lett* **66**, 2911-2914 (1991).

3 Krepelova, A., Newberg, J. T., Huthwelker, T., Bluhm, H. & Ammann, M. The nature of nitrate at the ice surface studied by XPS and NEXAFS. *Phys Chem Chem Phys* **12**, 8870-8880 (2010).

4 Wilson, K. R., Tobin, J. G., Ankudinov, A. L., Rehr, J. J. & Saykally, R. J. Extended x-ray absorption fine structure from hydrogen atoms in water. *Phys Rev Lett* **85**, 4289-4292 (2000).

5 Wilson, K. R. *et al.* X-ray spectroscopy of liquid water microjets. *J Phys Chem B* **105**, 3346-3349 (2001).

6 Wilson, K. R. *et al.* X-ray absorption spectroscopy of liquid methanol microjets: Bulk electronic structure and hydrogen bonding network. *J Phys Chem B* **109**, 10194-10203 (2005).
